# Cranial Osteogenesis and Suture Morphology in *Xenopus laevis*: A Unique Model System for Studying Craniofacial Development

**DOI:** 10.1371/journal.pone.0003914

**Published:** 2009-01-19

**Authors:** Bethany J. Slater, Karen J. Liu, Matthew D. Kwan, Natalina Quarto, Michael T. Longaker

**Affiliations:** 1 Division of Plastic and Reconstructive Surgery, Stanford University School of Medicine, Hagey Laboratory for Pediatric Regenerative Medicine, Stanford, California, United States of America; 2 Department of Surgery, Stanford University School of Medicine, Hagey Laboratory for Pediatric Regenerative Medicine, Stanford, California, United States of America; 3 Department of Craniofacial Development, King's College London, London, United Kingdom; 4 Department of Structural and Functional Biology, University of Naples Federico II Complesso M. S. Angelo, Napoli, Italy; American Museum of Natural History, United States of America

## Abstract

**Background:**

The tremendous diversity in vertebrate skull formation illustrates the range of forms and functions generated by varying genetic programs. Understanding the molecular basis for this variety may provide us with insights into mechanisms underlying human craniofacial anomalies. In this study, we provide evidence that the anuran *Xenopus laevis* can be developed as a simplified model system for the study of cranial ossification and suture patterning. The head structures of *Xenopus* undergo dramatic remodelling during metamorphosis; as a result, tadpole morphology differs greatly from the adult bony skull. Because of the extended larval period in *Xenopus*, the molecular basis of these alterations has not been well studied.

**Methodology/Principal Findings:**

We examined late larval, metamorphosing, and post-metamorphosis froglet stages in intact and sectioned animals. Using micro-computed tomography (μCT) and tissue staining of the frontoparietal bone and surrounding cartilage, we observed that bone formation initiates from lateral ossification centers, proceeding from posterior-to-anterior. Histological analyses revealed midline abutting and posterior overlapping sutures. To determine the mechanisms underlying the large-scale cranial changes, we examined proliferation, apoptosis, and proteinase activity during remodelling of the skull roof. We found that tissue turnover during metamorphosis could be accounted for by abundant matrix metalloproteinase (MMP) activity, at least in part by MMP-1 and -13.

**Conclusion:**

A better understanding of the dramatic transformation from cartilaginous head structures to bony skull during *Xenopus* metamorphosis may provide insights into tissue remodelling and regeneration in other systems. Our studies provide some new molecular insights into this process.

## Introduction

The mammalian skull vault is made up largely of paired bones that grow toward one another. Specialized joint structures called cranial sutures form between the developing bones. Proper ossification of the cranial bones and subsequent fusion of the cranial sutures provide protection for the central nervous system while allowing for expansion of the brain throughout childhood.[1] Deviation from the normal complex process of craniofacial development and morphogenesis results in various pathological congenital malformations. Although there has been progress made towards elucidating the events surrounding cranial ossification and suture fate, much of the biology remains poorly understood.[2] Improved knowledge of both the genetic events and molecular interactions dictating skull vault patterning and osteogenesis will likely provide insight into human craniofacial development and anomalies.

The majority of molecular studies on the development of the skull roof have drawn upon mutational analyses in murine models and genetic linkage in humans. In addition, studies of embryonic tissue contributions to the chondrocranium have been largely extrapolated from grafting experiments in chicks.[3], [4] More recently, large-scale genetic screens have been performed in zebrafish, and these are now beginning to identify mutants with craniofacial defects, although to date these screens have not focused on cranial suture formation.[5], [6], [7] Finally, evolutionary morphologists have compared cranial ossification sequences from divergent clades of tetrapods.[8] Although the architecture of these vertebrate skulls varies dramatically, there seems to be a surprising degree of conservation in the developmental machinery underlying pattern formation. In particular, the dermal bone comprising much of the skull roof appears very ancient. Evidence of these dermal bones has been found as far back as the agnathan fossil fishes.[9] Analysis of the development of mouse, chick, and zebrafish skull vault suggest cranial bones and sutures may be analogous to those in the human skull.[10], [11]

Although the early developmental stages of the South African frog, *Xenopus laevis*, have been described extensively[12] and *Xenopus* has been a model system for some aspects of craniofacial development, the details of calvarial osteogenesis, and particularly of cranial suture patterning, have not been fully investigated. Early descriptions of the chondrocranium were all based on examination of sectioned material and the illustrations were hand-drawn reconstructions.[13], [14] In 1992, Trueb and Hanken re-examined the skeletal development of *Xenopus* and showed that the frontoparietal is the first bone to differentiate in the skull, from roughly tadpole stage 54, and has fully ossified by stage 66.[15] More recently, using an indelible fluorescent dextran marker, several groups have shown that the entire frontoparietal bone in *X. laevis* and related frog *Bombina orientalis* is of neural crest derivation.[16], [17], [18] In the course of these studies, Gross *et al.* demonstrated the feasibility of long-term lineage studies of the neural crest in *Xenopus laevis*.[19] These kinds of experiments have historically been difficult because of the long larval period of *Xenopus*.[20] These advances, as well as the increasing availability of the genome from the closely related diploid frog, *Xenopus tropicalis*, led us to examine growth and ossification of the *Xenopus* cranium, as well as suture patterning and fate. Additionally, given that amphibians are evolutionarily positioned between primitive aquatic species and highly derived tetrapods, or amniotes,[20] investigating the process of cranial ossification can potentially increase our understanding of diverse cranial patterning as well as the evolution of animals such as mice and humans.

Our objective was to assess the feasibility of utilizing *Xenopus laevis* as an alternative model system for the study of cranial osteogenesis. We hypothesized that due to the unusual demands of metamorphosis, *Xenopus* would provide a fascinating example of developmental plasticity during a major developmental transition. In addition, tissue alterations during metamorphosis are similar to changes that occur in regeneration, remodelling, and wound healing. Thus, studying these transitions in *Xenopus* may prove to be useful for studying the regulation of tissue remodelling.

In this study we show that ossification of the frontoparietal bone of *Xenopus* initiates from lateral ossification centers and proceeds from posterior to anterior. Our observations confirm previous reports of the ossification sequence of *Xenopus*, with the frontoparietal ossifying earliest, followed by the parasphenoid.[15] Histological analyses revealed abutting, midline sutures. In addition, at the posterior aspect of the skull, we identified lateral overlapping sutures resembling murine lambdoid sutures. We document the post-metamorphosis morphology of these sutures. We then analyzed cellular proliferation, cellular apoptosis, and tissue turnover via matrix metalloproteinases within the skull roof during the stages of remodelling from the period of the cartilaginous structures found in larvae to the bony post-metamorphic skulls of froglets.

## Results

### *Xenopus* undergoes dramatic shape changes in the head region during metamorphosis

**Figure 1:**
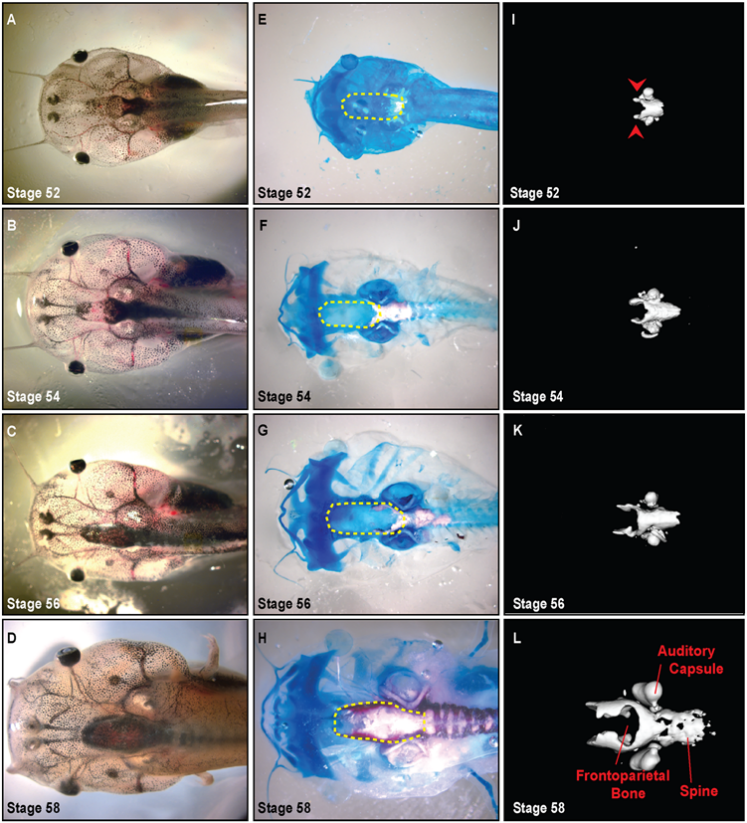
Development of the bony skull during pre-metamophosis (stages 52–58). A–D. Dorsal view of unstained *Xenopus* cranium demonstrates gross morphology, 0.7× magnification. E–H. Alizarin red (bone) and Alcian blue (cartilage) whole-mount staining of skull. Dashed yellow lines denote region of frontoparietal bone. I–L. μCT scans delineate ossified portions of the cranium. Note ossification proceeding from the base of the skull to the anterior aspect. Ossification of the frontoparietal bone begins at Stage 52 initially appearing laterally (arrow heads in panel I).

During metamorphosis, dramatic shape changes occur in the *Xenopus* head (**Figure 1** **,** **2** **,** **3**). Prior to metamorphosis, at stage 52 (staging according to Niewkoop and Faber[12]), the tadpole head is bulbous; the eyes are positioned laterally with the mouth parts protruding anteriorly (**Figure 1**). As metamorphosis progresses, the head narrows and shortens while the gill arches are being resorbed. By stage 66, the animal has adopted a more condensed triangular shape prefiguring the adult head (**Figure 3, C″**). Consistent with previous reports, we found that the larval head structures appeared largely cartilaginous (**Figure 1**) while in the post-metamorphosis frog, ossified bone makes up the vast majority of the head (**Figure 3**).[21]

**Figure 2 pone-0003914-g002:**
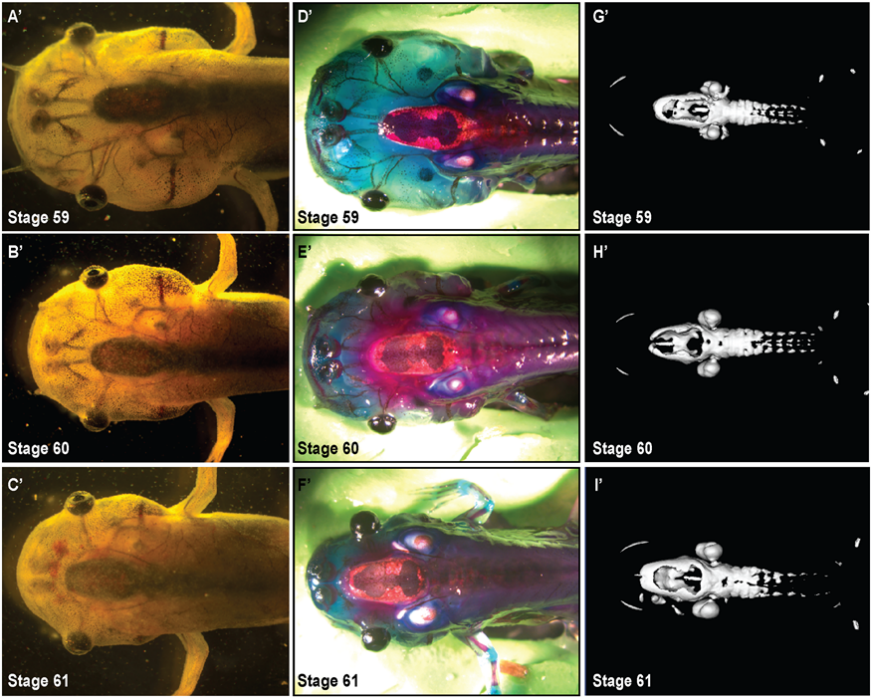
Development of the bony skull during metamorphosis (stages 59–61). A′–C′. Dorsal view of unstained *Xenopus* cranium demonstrates gross morphology, 0.7× magnification. D′–F′. Alizarin red (bone) and Alcian blue (cartilage) whole-mount staining of cranial skull. G′–I′. μCT scans delineate ossified portions of the cranium. Note ossification of the frontoparietal bone proceeding from the lateral aspect of the bone corresponding to the Alizarin red staining.

**Figure 3:**
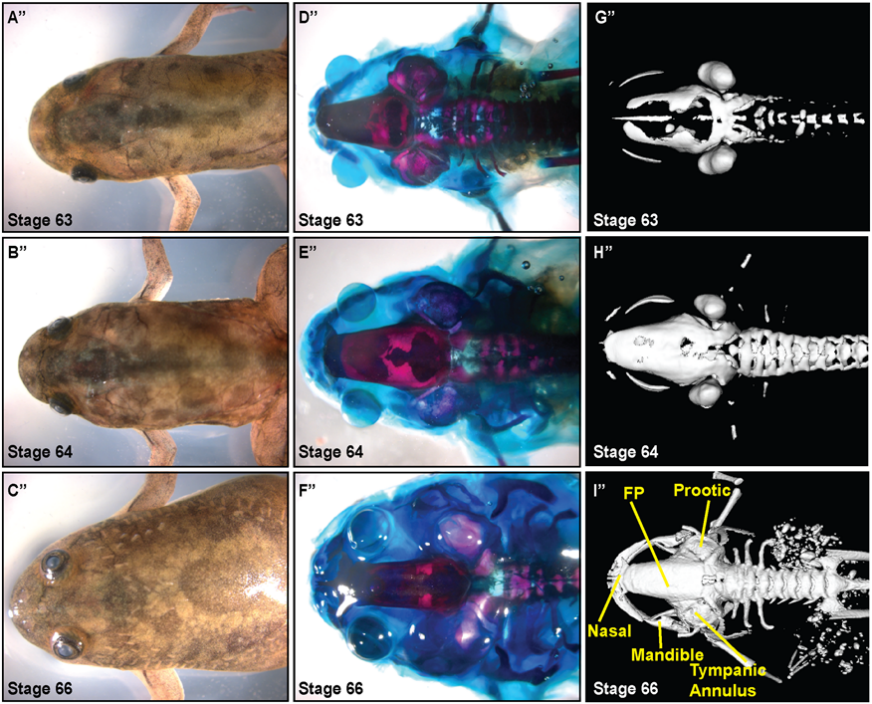
Development of skull after metamorphosis (stages 63 - frog). A″–C″. Dorsal view of unstained *Xenopus* cranium demonstrates gross morphology, 0.7× magnification. D″–F″. Alizarin red and Alcian blue whole-mount staining of cranial skull. G″–I″. μCT scans delineate ossified portions of cranium. Ossification is complete by Stage 64–66. Labelling based on Trueb and Hanken, FP: frontoparietal.

### Cranial ossification initiates laterally

Recent advances in micro-computed tomography (μCT) (reviewed in Ritman 2004)[22] have allowed fine resolution three-dimensional imaging of ossification in small animals. We took advantage of μCT technology to re-examine the ossification sequences of the metamorphic skull. We compared the three-dimensional reconstructions to stage-matched whole mount preparations stained for bone and cartilage (Alizarin red and Alcian blue, respectively) (**Figure 1**). The μCT analyses confirmed that the central frontoparietal bone was the first to appear. Onset of ossification is evident at Stages 52–54 (**Figure 1, I, J**). Prior reports suggested that the parasphenoid ossified soon after;[15], [23] however, we found that the otic capsules and axial column were the next to ossify. Based on our μCTs, parashenoid ossification was not evident until stage 59 (**Figure 2, G′**). As in amniotes, cranial ossification was initiated laterally (**Figure 1, I, arrowheads**). In *Xenpous*, this ossification then progressed from the caudal end of the skull to the anterior aspect. Ossification of the vertebrae began a stage later (stage 54–56), advancing from anterior to posterior (**Figure 1, F–K**).

### Morphogenesis of the frontoparietal bone

**Figure 4:**
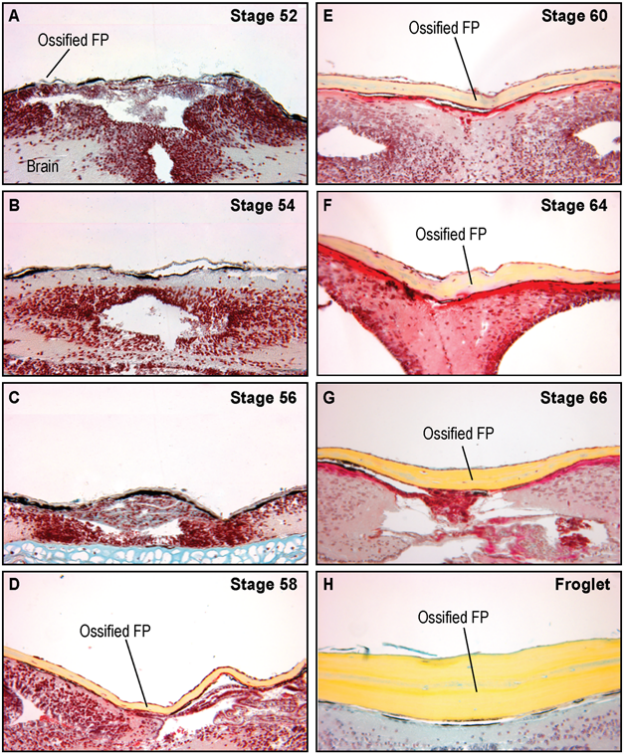
Frontoparietal sections. A–H. Coronal sections through the frontoparietal bone stained with Movat's pentachrome, 20× magnification. Ossification begins at Stage 52–54 and is completed by Stage 58–60. (yellow = bone, blue = cartilage, red = cytoplasm, dark red = osteoid) FP: frontoparietal.

We observed from the μCTs and whole mount bone preparations that ossification of the frontoparietal bone was initiated bilaterally and that the bones then extended toward the anterior aspect (**Figure 1**). As the tadpoles continued to develop from stages 59 to 61, ossification of the frontoparietal bone proceeded medially (**Figure 2**). Finally, in the post-metamorphosis stages, the frontoparietal bone continued to ossify until it formed one solid bone by stage 66 (**Figure 3**). At stage 66, the skull closely resembled the adult form. To investigate the morphogenesis of the frontoparietal bone in greater detail, we performed coronal sections through the head structures from stage 52 to froglet stages. We then stained these sections with Movat's pentachrome stain, which identifies cartilage (blue) and bone (yellow) (**Figure 4**). Histologically, bone formation began laterally and proceeded towards the midline from stages 52–58. (**Figure 4A–C, labelled ossified FP**). However, our observations did not distinguish between sequential ossification or expansion of the ossification field. By stage 58–60 the frontoparietal bone was completely ossified. (**Figure 4D–E**) Subsequent stages were characterized by thickening of the frontoparietal bone, with no evidence of a marrow cavity. (**Figure 4F–H**).

### Morphology of the sutures

**Figure 5:**
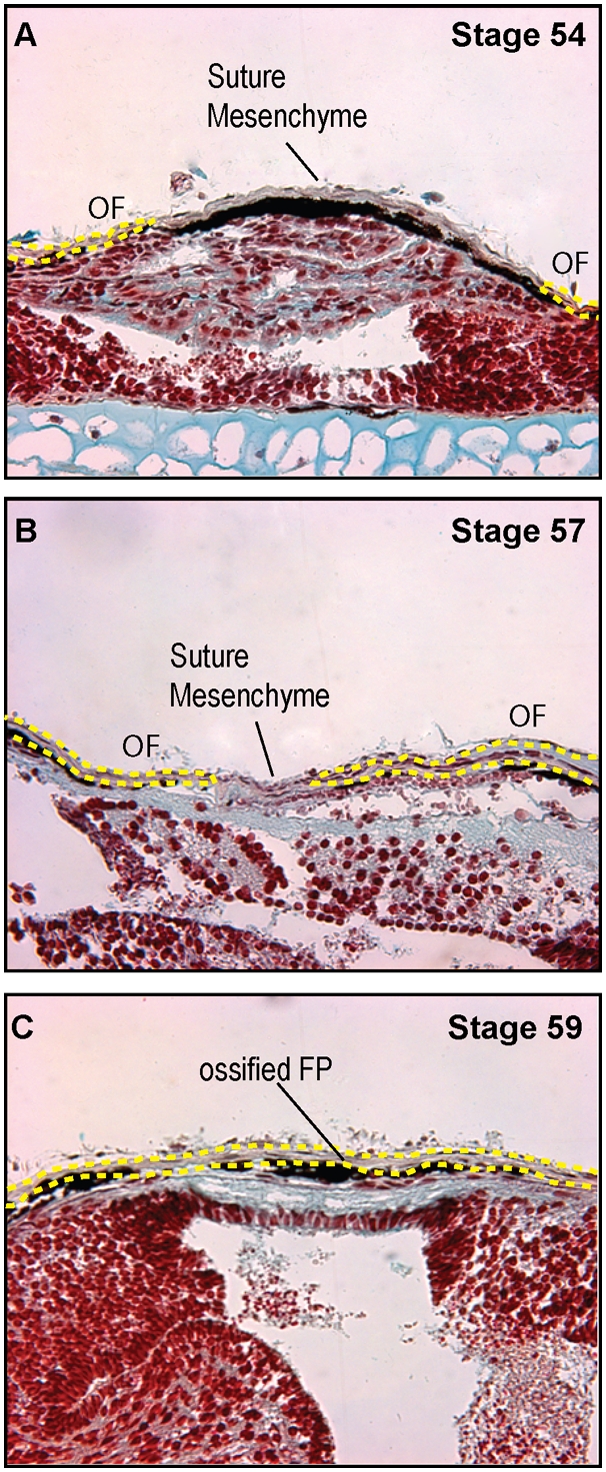
Anatomy of the midline suture. A–C. Movat's pentachrome stained coronal sections through the frontoparietal bone of A. Stage 54, B. Stage 57, and C. Stage 59, 40× magnification demonstrating midline, abutting sutures. Fusion of the suture occurs at Stage 58–60. OF: osteogenic front, FP: frontoparietal bone. The yellow dots indicate the margins of the osteogenic fronts and ossified bone.

Based on examination of the μCTs and whole mount bone preparations, we sought to determine whether an intermediate cranial suture, or joint, formed in the midline of the bone. Investigation of the coronal sections revealed an abutting, end-to-end suture in the midline in earlier stages. The suture fused between stages 58–60 forming a solid bone. (**Figure 5**)

**Figure 6:**
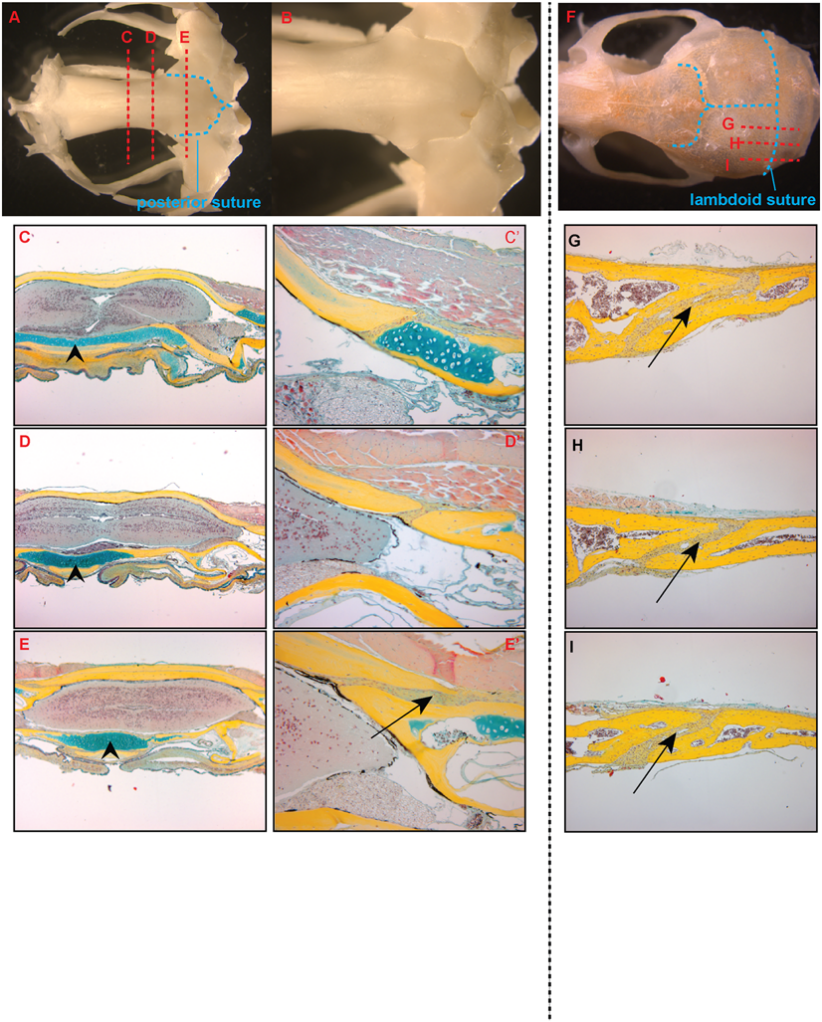
Morphology of the posterior suture. A. Dry skull preparation of adult *Xenopus* demonstrating the frontonasal and posterior sutures (outlined in blue dashed lines). B. Dorsal view of posterior suture at 1.25× magnification. C–E. Pentachrome stained coronal sections of an adult skull (5× magnification). The location of the planes of section are notated by red dashed lines in the dry skull prep in A. C′–E′. 20× magnification of suture. There are no midline sutures evident but at the posterior aspect, lateral overlapping sutures are present (Arrow in E′ note overlapping pattern). The skull base ventral to the brain has a cartilaginous layer throughout (arrowheads). F. Dry skull preparation of adult mouse demonstrating the lambdoid suture. G–I. Pentachrome stained parasagittal sections of adult mouse calvaria. Note the overlapping pattern of the suture (arrows).

In dry bone preparations as well as in our whole mount samples of adult *Xenopus* calvaria, we observed the existence of frontonasal and posterior sutures. Because we were interested in analyzing the anatomy of cranial sutures and comparing them to mammalian sutures, we examined the posterior suture in the adult frog. This suture was present at the posterior aspect of the skull where the frontoparietal bone overlaps the fused exoccipital and pro-otic bones (**Figure 6**). Serial coronal sections through the posterior aspect of the skull revealed histological evidence of sutures as well (**Figure 6C′–E′, arrow**). In these adult sections, the frontoparietal bone is clearly one solid block of calcified bone with only the posterior sections lying upon a cartilaginous cushion. This is in contrast to the skull base ventral to the brain, where throughout there is a cartilaginous layer adjacent to the parasphenoid bone (**Figure 6C–E, arrowheads**). The posterior sutures appear patent in these sections (**Figure 6E′, arrow**). In addition, the osteogenic fronts of the posterior sutures have an overlapping pattern that is similar in appearance to the murine lambdoid sutures (**Figure 6G–I, arrows**).

### Remodelling of the cartilaginous skull

Analyses of both the μCT images and the bone preparations confirmed the architectural changes in the developing *Xenopus* skull. The distance between the calcified mandible, first apparent as bilateral ossifications at stage 58–60, and the caudal skull is clearly diminishing (**Figure 2** **&** **3**). Concurrently, the position of the eyes shifted from the lateral binocular positions on the tadpole head to a more frontal position of the froglet (**Figure 1** **–** [Fig pone-0003914-g002] **3**). A large part of this remodelling appears to result from the dramatic loss of the cartilaginous tissues of the head in combination with the ossification of the bony skull.

**Figure 7:**
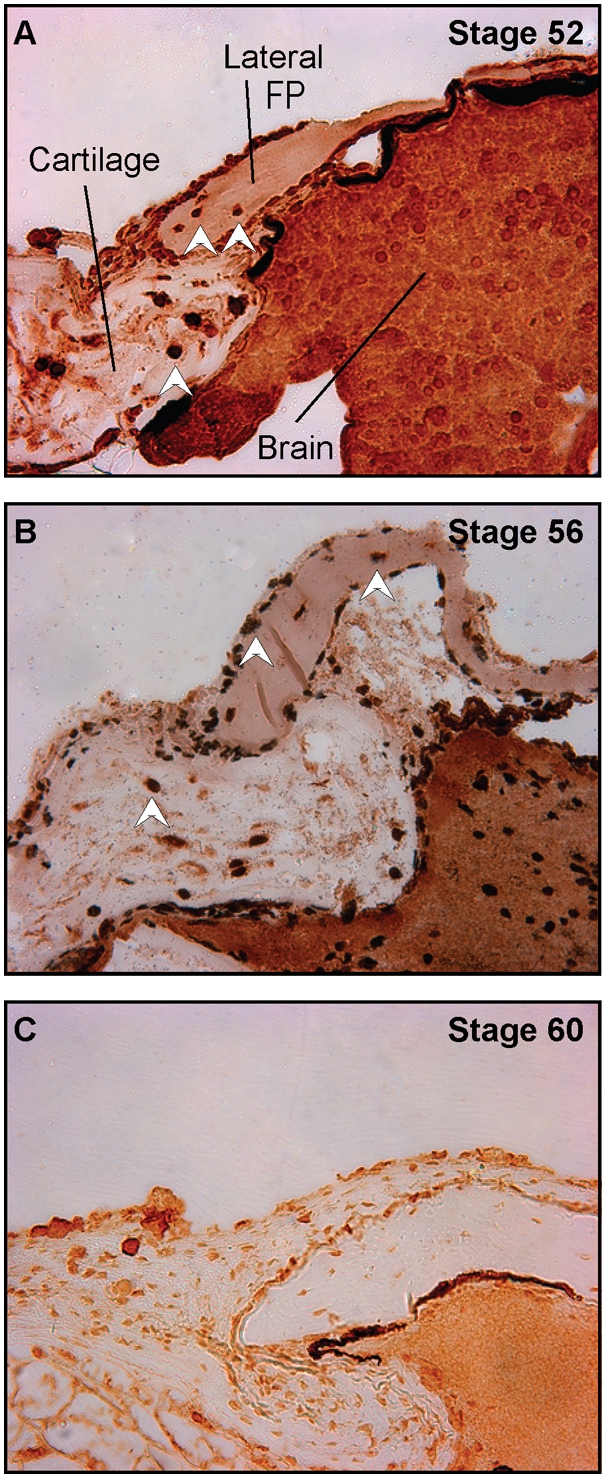
Proliferation during ossification of the skull. A–C. Phospho-Histone H3 staining of the frontoparietal bone. A. Stage 52, B. 56, and C. 60, 40× magnification. There is positive staining in the lateral aspects of the frontoparietal bone and surrounding cartilages at Stages 52 and 56 (white arrow heads); no staining is observed at Stage 60 once the frontoparietal bone has completely ossified.

We first investigated the role of proliferation in the remodelling process by examining the phosphorylation status of Histone H3, which marks dividing cells. While there was abundant proliferation in the lateral aspect of the frontoparietal bone and surrounding cartilage at earlier stages, this proliferation decreased significantly once the frontoparietal bone became completely ossified by Stage 58 (**Figure 7, arrowheads**).

We next examined the possibility that turnover of the cartilaginous skull was due to a developmental onset of cell death in the cartilage using TUNEL staining (data not shown). We examined sections through the frontoparietal bone during the period of greatest change (stages 52–64) and found no TUNEL positive cells in the bone among the various stages, although there were positive cells in the brain and in the surface ectoderm.

**Figure 8:**
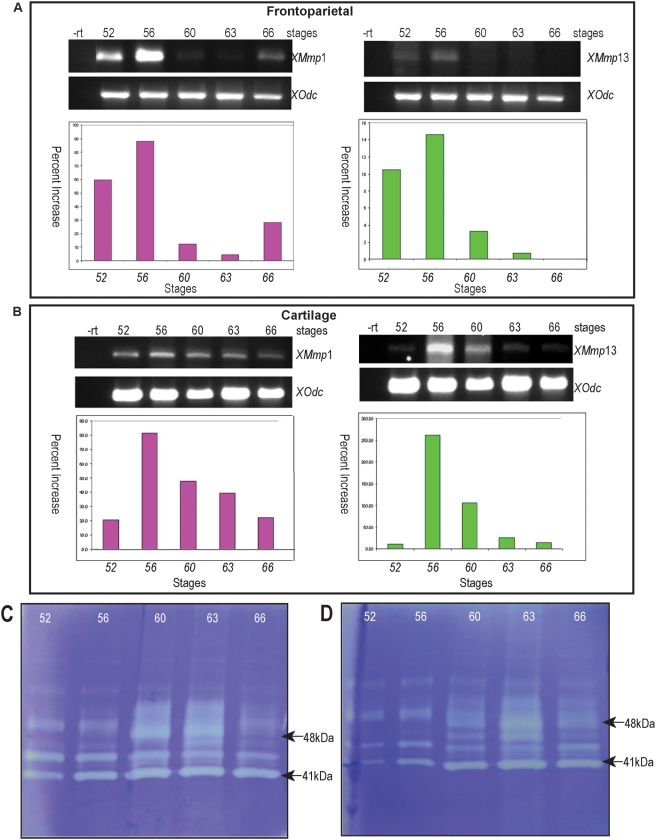
Expression of matrix metalloproteinases MMP-1 and MMP-13. A–B. semi-quantitative quantitative PCR. A. MMP-1 and -13 gene expression in the frontoparietal region. B. MMP-1 and -13 gene expression in the surrounding cartilage. Tissues were dissected at the indicated stages and semi-quantitative RT-PCR for MMP-1 and -13 was performed at indicated stages and normalized to expression of the housekeeping gene ornithine decarboxylase (odc). Note the peaks of expression correlating with stages with abundant tissue turnover. -rt: no reverse transcriptase control. C–D. Stage-specific gelatinase activity. Tissue samples from frontoparietal bone (C) and surrounding cartilage (D) were dissected at the indicated stages, concentrated and subjected to gelatin zymography. The clear bands represent proteinase activity. Major bands at 41 and 48 kDA correspond to the molecular weight of MMP-1 and MMP-13 respectively. Note the peak of lytic activity appears at a later stage than the peak of gene expression with PCR.

Finally, we examined the expression of matrix metalloproteinase (MMPs) genes in tissue turnover observed in the *Xenopus* cranium. MMPs are a large family of proteinases that cleave and remodel extracellular matrix.[24] We chose to focus on MMP expression and activity in the calvaria given that MMPs are known to be important in remodelling tissues during amphibian metamorphosis, particularly during intestinal development and tail and gill resorption.[25], [26] MMPs have also been implicated in the remodelling of mammalian cartilages into bone.[27] We first performed semi-quantitative PCR on the frontoparietal bone (**figure 8A**) and surrounding cartilage (**figure 8B**) at various stages in order to investigate the gene expression of MMP-1 and -13. There appeared to be high levels of expression in both the frontoparietal bone and surrounding cartilage during the times of the most active remodelling as evidenced by the peaks in the graphs. (**Figure 8**)

In order to assess MMP activity during the same stages, we used gelatin zymography (**Figure 8, C–D**). The clear bands in this figure represent lytic activity. Analogous to the PCR data, we found abundant MMP activity during the stages in which significant remodelling was occurring. The peak of activity was delayed by several stages compared to that found with PCR; this might be due to translational and post-translational processing of the MMPs.[28] The most intense activity appeared at the band migrating at approximately 41 kD, corresponding to the molecular weight of MMP-1. The bands which were the second most intensely staining migrated at approximately 48 kD, corresponding to the molecular weight of MMP-13.

**Figure 9:**
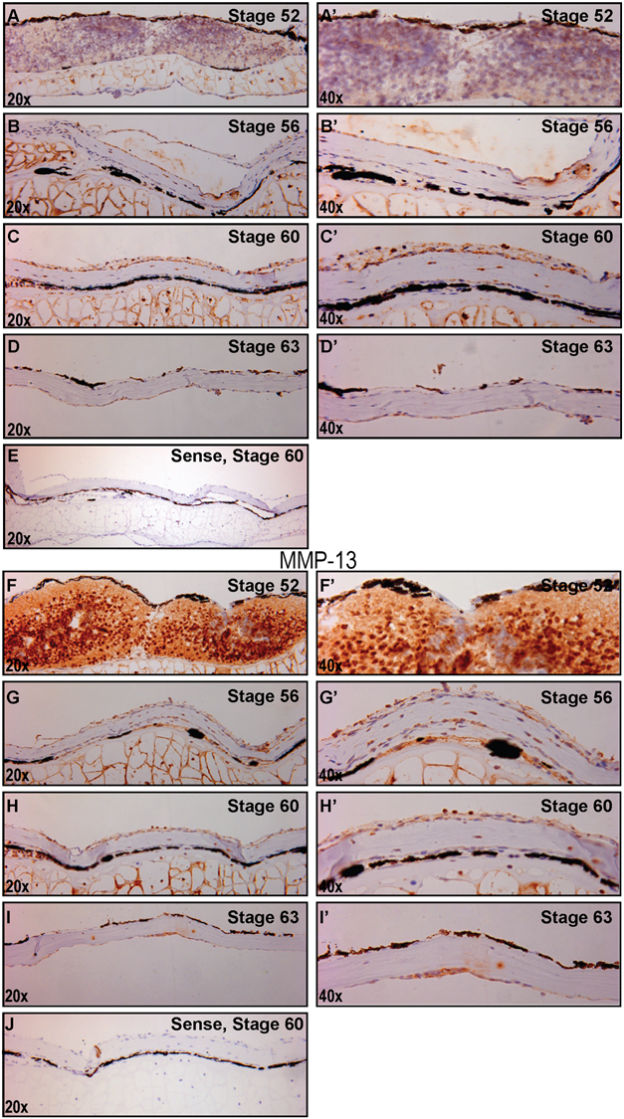
mRNA expression of MMP-1 and MMP-13. A–D. MMP-1 *In situ* hybridization of sections of frontoparietal bone from various stages (antisense probes), 20× magnification. A′–D′. 40× magnification of same area. E. Representative sense probe of stage 60 to demonstrate lack of non-specific staining. Brown staining represents positive localization, blue represent hematoxylin counterstaining. F–I. MMP-13 *In situ* hybridization of sections of frontoparietal bone from various stages (antisense probes), 20× magnification. F′–I′. 40× magnification of same area. J. Representative sense probe of stage 60. Note increased brown staining in Stage 56 and 60 with both probes.

Lastly, we performed *in situ* hybridization on the frontoparietal bones at the various stages to look more closely at the temporospatial relationship of MMP expression. Again, we observed a similar temporal pattern of increased MMP-1 and -13 expression (in brown) during the stages of the most dramatic remodelling. (**Figure 9**) We found abundant mRNA expression in the cartilage at stages 56 and 60 and a marked decrease by stage 63. These data confirm the gene expression and MMP activity findings. (**Figure 8**)

## Discussion

The development of the skull is a complex process controlled by multiple genetic and environmental factors.[1] Variation of skull patterning provides the animal kingdom with tremendous external diversity and functional adaptation. Because changes in bone formation and growth can lead to pathological consequences in humans, we wanted to better understand the developmental processes that lead to the manifestation of different cranial phenotypes. The frog, *Xenopus laevis*, provides a unique opportunity to study very specialized cranial structures in a well-characterized model system.[29] The cranial vault of *Xenopus* has been described but osteogenesis and suture morphology have not been systematically detailed. We have set out to document this process during metamorphosis, a period of substantial cranial remodelling.

In this study, we found that the *Xenopus* calvarium undergoes significant changes in its morphology during the time of cranial osteogenesis. Although *Xenopus* tadpoles go through a protracted larval state, the ossification of the frontoparietal bone, the major constituent of the skull, takes place from approximately stage 54 to 64 and initiates from lateral ossification centers. This pattern of ossification proceeding laterally to medially is similar to that described in other species, including mice, rats, and humans. In these species, the skull vault develops from mesenchymal cells that differentiate and deposit extracellular matrix. Intramembranous ossification then progresses medially from these foci.[1], [30] In addition, we found that ossification occurs in the posterior to anterior direction. Midline, abutting sutures appear and fuse by stage 58–60, as well as transversely-oriented, posterior sutures with an overlapping pattern, similar to the murine lambdoid suture.

Contrary to prior reports of ossification sequences in pipoid frogs as well as fish such as zebrafish, we found that, by μCT, the frontoparietals began ossifying prior to the parasphenoid.[31], [32], [33] It is possible that historical reports relied only on histological stains and visual interpretation whereas tomographic analysis only revealed fully mineralized regions. These differences will need to be resolved in future studies.

In the mammalian calvaria, there are two midline sutures, the interfrontal (or posterior frontal) and sagittal, and two transverse sutures, the coronal and lambdoid. The cranial sutures serve as joints permitting skull deformation and growth during periods of rapid brain expansion, particularly in early life.[1] The suture pattern of *Xenopus* is different than that of mammalian or avian species, primarily due to the fact that the frontal and parietal bones are fused, making up one bone in this species.[34] Ossification also occurs rapidly, over the course of two to three weeks. As such, cranial anatomy is simpler in *Xenopus* when compared to humans. *Xenopus* has only two sutures, a midline suture which fuses prior to metamorphosis and a posterior suture that remains patent. We speculate that the early fusion of the midline sutures in *Xenopus* is most likely due to the lack of expansion of the brain alleviating the necessity for continued growth centers. In addition, the lack of rapid forebrain expansion may also explain the comparatively rapid ossification of the frontoparietal bone. It is not clear from our data whether the posterior suture in *Xenopus* is homologous to that of mouse or human: further molecular characterization will be necessary. Nevertheless, sutural interactions (resulting in patency or fusion) may be analogous. Again, further analysis will likely shed light on these issues.

In the remaining skeleton, we found that by μCT scanning, ossification of the vertebrae became evident after that of the skull (**Figure 1** **–** [Fig pone-0003914-g002] **3**). This is consistent with previous reports of dermal bone development preceding that of endochondral bones in pipoids as well as in amniotes.[32], [35], [36]

We were also interested in the tremendous tissue turnover that occurs in *Xenopus* during cranial development. Cartilaginous replacement occurs over the stages investigated in this study, and we chose to examine levels of proliferation and apoptosis in tissue turnover. We did note abundant lateral proliferation of the frontoparietal bone in the pre-metamorphosis stages; however, this proliferation was markedly decreased by the onset of frontoparietal bone ossification. In addition, we found no significant increases in TUNEL staining among the stages, indicating that the remodelling was unlikely due to apoptosis, although we could not exclude the possibility of alternative mechanisms of cell death.

MMPs are a family of proteases involved in the degradation and remodelling of extracellular matrix. Collagenolytic activity was first described from studies on the anuran tadpole tail.[37] Subsequently, both MMP-1 and -13 have been reported to be expressed in the resorbing tail during metamorphosis[25], [26], [38], however, the cranial cartilage has not been examined. In addition, in mammals, MMP1 has been implicated in coordinating cartilage dissolution during cranial ossification.[39] These authors found that deficiency of MMP-1 in mutant mice results in severe skeletal pathology, further demonstrating the importance of remodelling of cartilage for skeletal growth. MMP-13-null mice also show skeletal and cartilage abnormalities.[40] Our data suggest that remodelling of the *Xenopus* skull is largely associated with tissue turnover via MMP activity, at least in part by MMP-1 and MMP-13. Our results are similar to Holmbeck *et al.*'s findings in mice that a spatially and temporarily restricted expression of MMPs leads to removal of a calvarial cartilage anlagen.[39] However, while they found abundant apoptosis during tissue turnover in mouse, we did not detect an increase in apoptotic cells at this stage. Therefore, we could not rule out the possibility that *X. laevis* cranial chondrocytes persist. This scenario would suggest that MMP-mediated restructuring of the cartilaginous matrix is important for remodelling of the head; quiescent chondrocytes could then remain behind, leaving the potential for re-activation in needed.

*Xenopus laevis* has long been used as a model system for developmental studies. Manipulation of the embryos is straightforward, and many molecular tools and reagents are available. However, very little is known about the development of the skull. Our studies demonstrate the feasibility of tracking cranial osteogenesis in *Xenopus* and provide the groundwork for future molecular analyses. In addition, the genome of a related diploid species, *Xenopus tropicalis*, has recently become available. Based on our observations, the development of the adult *X. tropicalis* cranial architecture appears quite similar to that of *X. laevis* (data not shown) and others have begun to study molecular regulation of skeletogenesis in *X. tropicalis*.[41] Several groups have begun identifying genetic mutants in *X. tropicalis*, and we anticipate that they will identify mutants with craniofacial phenotypes. As these resources become widespread, we hope to be able to identify novel conserved mechanisms of tissue remodelling in the skull.

## Materials and Methods

### Developmental Staging

*Xenopus laevis* tadpoles were obtained from Nasco and staged according to Nieuwkoop and Faber.[12] The tadpoles were grouped into pre-metamorphosis (Stages 52–58), metamorphosis (Stage 58–61), and post-metamorphosis (Stage 63-froglet) stages. The tadpoles were then inspected for observable morphological changes in the skull among the stages. The Stanford University Animal Care and Use Committee (ACUC) and Administrative Panel on Laboratory Animal Care (APLAC) approved all animal care procedures.

### Tissue Staining

Tadpoles from Stages 52–66 (three to five tadpoles per stage) were collected and euthanized by immersion in anaesthetic (0.05% benzocaine in H_2_0), fixed for one hour in MEMFA, and washed several times in phosphate-buffered saline. In order to characterize skull development, whole-mount preparations were performed on all of the tadpole stages. For cartilage detection, specimens were stained with 0.01% Alcian blue for two nights and destained with 100% alcohol followed by 1% potassium hydroxide (KOH). For bone detection, the specimens were stained with 0.02% Alizarin red and 1% KOH overnight at room temperature. All specimens were cleared in graded glycerol for 2–5 days. The specimens were analyzed under a dissecting microscope, and images were acquired using a Leica MZ16 equipped with a DC500 digital camera.

### Micro-computed Tomography (μCT)

For three-dimensional imaging of ossification, we used μCT to evaluate the calcified portions of the skull. After euthanasia, a representative specimen from each stage was selected for μCT scanning at a 45 µm resolution (μCT, eXplore RS Micro CT System, GE Healthcare, United Kingdom). The scans were reconstructed as three-dimensional isosurfaces using Microview software (GE Healthcare) and compared to Alizarin red staining.

### Histological sections

To track morphogenesis of sutures, we performed histological analyses from representative skulls isolated from the three groups, pre-metamorphosis, metamorphosis, and post-metamorphosis stages. The specimens were fixed in MEMFA for one hour and decalcified with 19% ethylenediaminetetraacetic (EDTA) acid at 4°C for 2–7 days. The specimens were then serially dehydrated in alcohol and embedded in paraffin. Five micron histological sections were obtained in the coronal plane using a microtome (Micro HM-320) and mounted on Superfrost plus slides (Fisher Scientific). Every 10th to 15th slide was stained with Movat's pentachrome for bone and cartilage identification. The sections were analyzed and photographed with a Zeiss Axioplan 2 microscope equipped with an Axiocam HRc digital camera.

### Dry Bone Prep

An adult male skull was subjected to dry skull preparation using a beetle colony in order to evaluate gross suture morphogenesis and calvarial anatomy. The skin was removed from the skull of the euthanized animal and placed in a beetle colony for one day for removal of soft tissue. After all of the tissue had been removed, the skull was treated with 70% alcohol, allowed to dry, and then bleached using 3% hydrogen peroxide.

### Immunohistochemistry

For mitosis identification, phospho-Histone H3 antibody staining on histological sections was performed as previously described.[42] Sections of previously MEMFA-fixed tissue were deparaffinized, rehydrated, and treated with citrate buffer for epitope retrieval. The slides were then incubated with 1% hydrogen peroxide to block any endogenous peroxidase activity and stained with anti-phospho-histone H3 antibody (Upstate) at a 1∶200 dilution. After rinsing with phosphate buffered saline (PBS), biotinylated goat anti-rabbit IgG secondary antibody (Vector Laboratories) was applied and detected using HRP-streptavidin at a 1∶10,000 dilution (Jackson ImmunoResearch Laboratories) and revealed using a DAB kit (Zymed Laboratories) Sections were then counterstained with DAPI for immunofluorescent nuclear recognition.

TUNEL staining (*In Situ* Cell Death Detection Kit, Fluorescein, Roche) was performed on adjacent histological sections at various stages for detection of apoptosis. The slides were counterstained with DAPI for nuclear identification. Positive controls were treated with DNaseI prior to the labelling procedure to induce double strand DNA breaks, while negative controls were incubated without the reaction mixture.

### RNA Isolation and Reverse Transcription-PCR Analysis

Total RNA was isolated from fresh tissue dissected from the frontoparietal region and surrounding cartilage of the various stages. The tissue was then homogenized using a pellet pestle motor (Kontes). Purified and quantified RNA was treated with DNAse I (Ambion; Austin, TX) to clear genomic DNA. Two micrograms of total RNA was then reverse transcribed to cDNA using random hexamer primers (Invitrogen, Carlsbad, CA). Reverse transcription was performed for 1 hour at 42°C, followed by incubation at 75°C for 5 minutes to inactivate reverse transcriptase. RNAse treatment was used to clear residual RNA. PCR reactions were performed under the following conditions: 94°C for 5 minutes, 94°C for 30 seconds, annealing for 1 minute, and 72°C for 1 minute (25–35 cycles). Specific primers for the genes examined were designed based on their GenBank sequence. Primer sequences for the genes are listed below. For the densitometric analysis of RT-PCR, bands were scanned and quantified by using the ImageJ program 1.36b, (National Institutes of Health, Bethesda, MD). The densitometric results of each band was normalized to their respective loading control (*ornithine decarboxylase: odc*) and presented as relative levels.

**Oligos:** ***XMmp1*** **Forward:** ACGTGGCCAATTTCAGAGTTTTC;
***XMmp1*** **Reverse:** CATCAGGGCATTAGGGTCATTAGA;
***XMmp13*** **Forward:** TGCCGCGACCTTCATCAGA;
***XMmp13*** **Reverse:** AAAGCCCCAGGTCCAGAGTATTC;
***XOdc*** **Forward:** AGCGCTCCCCCGTGTCA;
***XOdc*** **Reverse:** CTCAGCCCCCATGTCAAAGA

### Gelatin Zymography

Calvaria (stages 52, 56, 60, 63, and 66) were dissected, placed into lysis buffer (10 mM Tris, pH 7.5, 0.1% Triton-X), and mechanically homogenized. The amount of protein obtained was quantified using the BCA protein assay (Pierce). An equal amount of protein from the different samples was added to Gelatin Sepharose 4B beads (Amersham) and allowed to mix for 2 hours at 4°C in order to concentrate the protein. The protein samples and beads were electrophoresed on a 10% gelatin zymogram gel (Bio-Rad Laboratories). The gels were then renatured to allow the proteases to break down the substrate contained in the gel and developed according to the manufacturer's instructions. The lytic activity was detected as a clear band on a blue background after staining with Coomassie blue.

### *In Situ* Hybridization

Tissue sections were deparaffinized with xylene and rehydrated in graded ethanol. Endogenous peroxide activity was quenched with 0.3% H_2_O_2_ in DEPC H_2_O for 5 min and followed by two washes in DEPC H_2_O and once in TBS (BioRad, Hercules, CA). The sections were then incubated in proteinase K for 10 min at 37°C, washed with TBST (DakoCytomation), and pre-hybridized with hybridization solution (Dako) at 56°C for 1–2 hr. Hybridization was then performed with MMP-1 and-13 sense and anti-sense probes overnight at 56°C. The sections were washed with SSC, RNAse treated (Ambion, diluted 1/35 in SSC), and washed with sequential dilutions of SSC. Biotin blocking was performed with DAKO Biotin Blocking system. Next, blocking was performed with blocking solution for 30 min at room temperature (Roche) and rabbit immunoglobin fraction (Dako). Rabbit HRP-anti-Ddig antibody (Dako, 1∶100) was used for 2 hr. After washing, biotnyl-tyramide and secondary streptavidine for 15 min were added (Dako GenPoint kit). Color was detected using 3,3′ diaminobenzidine (DAB) solution according to the manufacturer's directions. Hematoxylin was used for counterstaining.
